# CD59 Protects Primary Human Cerebrovascular Smooth Muscle Cells from Cytolytic Membrane Attack Complex

**DOI:** 10.3390/brainsci14060601

**Published:** 2024-06-14

**Authors:** Carson D. Whinnery, Ying Nie, Danilo S. Boskovic, Salvador Soriano, Wolff M. Kirsch

**Affiliations:** 1 Division of Biochemistry, Department of Basic Sciences, School of Medicine, Loma Linda University, Loma Linda, CA 92350, USA; cwhinnery@students.llu.edu (C.D.W.); dboskovic@llu.edu (D.S.B.); wkirsch@llu.edu (W.M.K.); 2 Neurosurgery Center for Research, Training and Education, School of Medicine, Loma Linda University, Loma Linda, CA 92350, USA; ynie@llu.edu; 3 Laboratory of Neurodegenerative Diseases, Department of Pathology and Human Anatomy, School of Medicine, Loma Linda University, Loma Linda, CA 92350, USA

**Keywords:** CD59, complement, cytotoxicity, membrane attack complex, innate immunity, human cerebral vascular smooth muscle

## Abstract

Cerebral amyloid angiopathy is characterized by a weakening of the small- and medium-sized cerebral arteries, as their smooth muscle cells are progressively replaced with acellular amyloid β, increasing vessel fragility and vulnerability to microhemorrhage. In this context, an aberrant overactivation of the complement system would further aggravate this process. The surface protein CD59 protects most cells from complement-induced cytotoxicity, but expression levels can fluctuate due to disease and varying cell types. The degree to which CD59 protects human cerebral vascular smooth muscle (HCSM) cells from complement-induced cytotoxicity has not yet been determined. To address this shortcoming, we selectively blocked the activity of HCSM-expressed CD59 with an antibody, and challenged the cells with complement, then measured cellular viability. Unblocked HCSM cells proved resistant to all tested concentrations of complement, and this resistance decreased progressively with increasing concentrations of anti-CD59 antibody. Complete CD59 blockage, however, did not result in a total loss of cellular viability, suggesting that additional factors may have some protective functions. Taken together, this implies that CD59 plays a predominant role in HCSM cellular protection against complement-induced cytotoxicity. The overexpression of CD59 could be an effective means of protecting these cells from excessive complement system activity, with consequent reductions in the incidence of microhemorrhage. The precise extent to which cellular repair mechanisms and other complement repair proteins contribute to this resistance has yet to be fully elucidated.

## 1. Background

Cerebral amyloid angiopathy (CAA) is a vascular pathology characterized by the thickening of small and medium arteries in the brain due to the deposition of amyloid β (AB) within the media and adventitia of vessel walls. Over time, the smooth muscle (SM) tissue is replaced with acellular AB plaques, accompanied by the development of structural fragility and vulnerability to rupture [1]. Alternatively, severe cases of microvascular AB deposition are also associated with cerebral microinfarcts [2]. CAA pathology is associated with several diseases, and is estimated to coincide with 75–90% of all cases of Alzheimer’s disease [1,3,4,5]. Cerebrovascular dysfunction is now recognized as a risk factor for the onset of dementia. CAA-induced microhemorrhages may contribute to the progression of dementia by increasing the amount of iron present in the brain, resulting in oxidative damage and neurodegeneration [1,6,7,8]. By one statistical estimate, the average contribution of CAA to the total cognitive decline of afflicted individuals is 15.7% [5]. No effective treatment for CAA currently exists [9,10].

**Figure 1:**
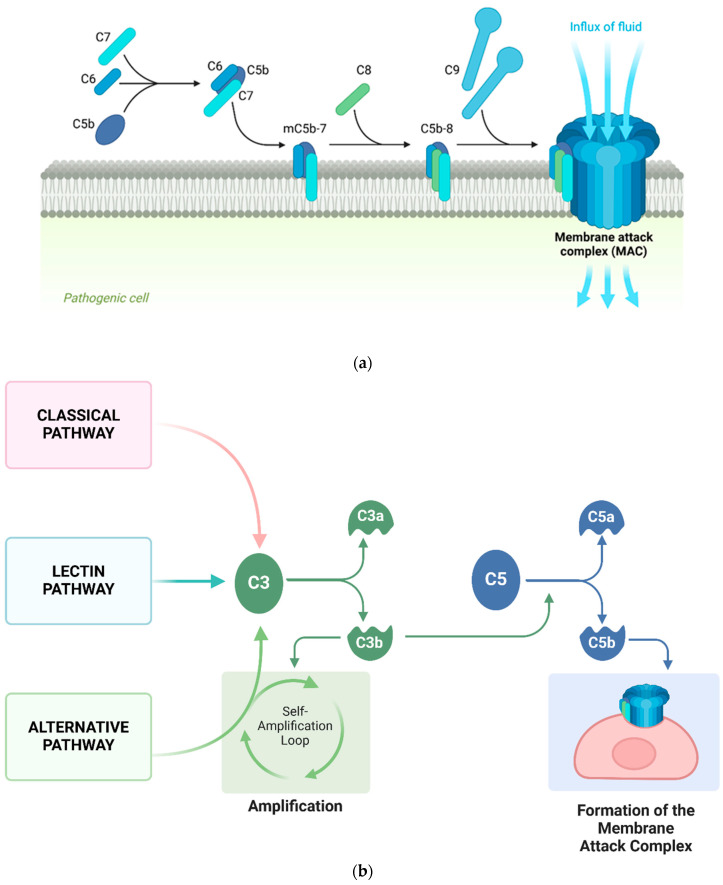
Simplified illustrations of the formation of the membrane attack complex (MAC) and operation of the complement system. (**a**) Complement protein fragment C5b complexes with complement proteins C6, C7, C8, and multiple molecules of C9 to form a functional membrane attack complex (MAC) in the surface membrane of pathogens. The MAC forms a channel between the extracellular and intracellular environments to promote cytolysis. (**b**) Simplified illustration of the complement system. The protein cascade is initiated through antigen–antibody complexes (classical pathway), the recognition of conserved pathogenic carbohydrate motifs (lectin pathway), or the spontaneous hydrolysis of the C3 complement protein (alternative pathway). The three pathways converge at the hydrolysis of C3 into C3a and C3b protein fragments. C3b assembles with other complement factors to promote additional C3 hydrolysis, forming a self-amplification loop. C3b also incorporates into convertases, which cleave the C5 protein to form a C5b protein fragment, which goes on to assemble with other complement proteins to form the membrane attack complex (MAC). The illustrations were adapted from “Formation of the Membrane Attack Complex” and “Roles of the complement Cascade in Innate Immunity” by BioRender.com (2024). Retrieved from https://app.biorender.com/biorender-templates, accessed on 8 March 2024.

Our prior work suggests a role for the membrane attack complex (MAC) in the progression of CAA [3]. The MAC, also referred to as C5b-9, is an assembly of plasma proteins that, together, form a trans-membrane pore structure directly connecting the internal and external cellular environments (Figure 1a). The resultant unrestricted osmotic exchange initiates cytolysis [11,12,13]. MAC formation is a product of the complement cascade, a network of protein interactions comprising three recognized pathways (classical, alternative, or lectin), with their distinct activation mechanisms (Figure 1b). In simplified terms, the classical pathway is activated by antigen–antibody complexes, the alternative pathway can be activated spontaneously, and the lectin pathway is activated via the recognition of conserved pathogenic carbohydrate motifs [14]. The complement cascade is an aspect of the innate immune response and mediates a number of cellular functions. Its convergent cytolytic endpoint, the MAC, plays an important role by damaging and eliminating susceptible pathogenic microorganisms [15,16,17]. Human cell membranes are likewise vulnerable to the cytolytic activity of MAC.

**Figure 2:**
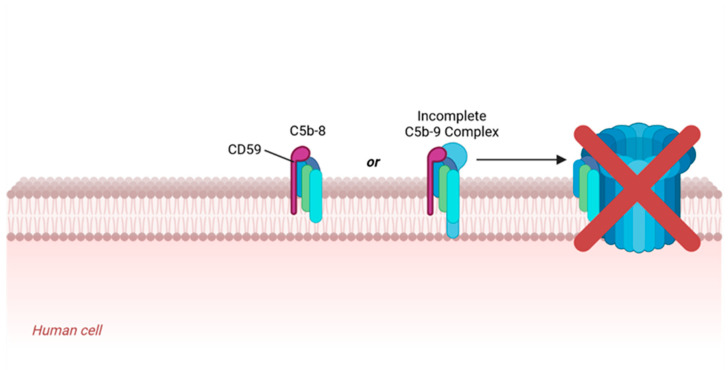
Simplified illustration of CD59 inhibiting formation of the membrane attack complex. CD59 molecules expressed by human cells bind to C5b-8 or incomplete C5b-9 complexes and block the incorporation of multiple C9 molecules, preventing the formation of functional MACs. The illustration was adapted from “Formation of the Membrane Attack Complex” by BioRender.com (2024). Retrieved from https://app.biorender.com/biorender-templates, accessed on 8 March 2024.

To prevent host cell damage, a system of surface membrane-bound and fluid-phase complement regulatory proteins intervene at a number of steps in the complement cascade to prevent aberrant MAC formation. One of the most important of these regulators is the glycophosphatidylinositol-anchored surface protein CD59, also referred to as protectin [18,19,20,21,22,23]. If an incomplete MAC complex (C5b-8) is inserted into the host cell membrane, and even after the incorporation of a C9 molecule, a functional CD59 protein can bind such a complex and inhibit the further full C9 polymerization (Figure 2). Consequently, the formation of MAC’s characteristic transmembrane pore structure is prevented, thereby preserving cellular surface membrane integrity [24,25,26,27]. While incidental MAC insertions may still occur and cause some membrane damage, such damage remains sub-lytic due to effective cellular repair mechanisms [28]. Two additional surface membrane-bound complement regulators include CD46 and CD55. CD46 serves as a cofactor enabling the inactivation of C3b and C4b complement proteins, while CD55 disrupts the C3 and C5 convertases necessary for the complement cascade to proceed [29].

Vulnerability to complement-mediated cytolysis can vary across cell types according to their levels of CD59 surface expression [11,25]. Low CD59 expression can sensitize cells to complement damage, while high expression confers resistance [20,23,30]. Normal expression levels of CD59 vary with tissue location, but can also fluctuate under abnormal conditions, such as Alzheimer’s disease, organ transplantation, or cancer [18,20,22,30,31,32]. Though CD59′s protection against complement was initially believed to be species-specific, later evidence demonstrated that some cross-species protection does occur [25,33,34,35]. A number of reports documented the decreased protective potential of CD59. Genetic mutations can compromise CD59′s anti-MAC functionality [30,36]. Insufficient CD59 expression is, or is believed to be, associated with a number of conditions, including paroxysmal nocturnal hemoglobinuria, Alzheimer’s disease, age-related macular degeneration, post-transplant organ rejection, and genetic demyelinating neuropathy in some patients [18,30,37,38,39,40,41,42]. Lower CD59 expression in the intracranial artery is associated with complement activation, inflammation, and the possible weakening of the arterial wall [43].

Earlier work suggested that CAA-afflicted cerebral blood vessels have increased MAC deposition without a compensatory upregulation of surface CD59 [3]. Over time, the cumulative cytotoxic and cytolytic damage could play a role in the gradual characteristic destruction of human cerebral vascular smooth muscle cells (HCSM). However, this potential sensitivity of primary HCSM cells to complement attack has not yet been reported. In this study, the primary HCSM cells were isolated from small blood vessels of the brain, obtained during routine temporal lobe biopsies. Their surface-expressed CD59 proteins were then inhibited in a controlled dose-dependent manner, in order to evaluate the role of CD59 in cellular resistance against complement-dependent cytotoxicity.

## 2. Materials and Methods

CD59 antibody MEM-43 (Abcam Cat# ab9182, RRID:AB_307053), desmin antibody Y66 with Alexa Fluor 594 (Abcam Cat# ab203419, RRID:AB_2943480), anti-α-smooth muscle actin antibody EPR5368 (Abcam Cat# ab124964, RRID:AB_11129103), secondary antibody with Alexa Fluor 594 (Abcam Cat# ab150116, RRID:AB_2650601), and secondary antibody with Alexa Fluor 488 (Abcam Cat# ab150077, RRID:AB_2630356) were purchased from Abcam (Cambridge, UK). Normal human serum was purchased from Cedarlane (Burlington, NC, USA). Collagen type-1-coated 96-well flat-bottomed plates were purchased from Fisher Scientific (Waltham, MA, USA). Rabbit red blood cells were purchased from Innovative Research Inc. (Novi, MI, USA). Smooth muscle cell media, FBS, penicillin/streptomycin, and SM cell growth supplement were purchased from ScienCell Research Laboratories (Carlsbad, CA, USA). Gelatin veronal buffer, glycerol, and Triton X were purchased from Millipore Sigma (Burlington, MA, USA). BSA, DPBS with calcium and magnesium, flat-bottomed 96-well plates, glass coverslips (12 mm) coated with rat tail-derived collagen-1, AlamarBlue HS resazurin cell viability reagent, CD59 antibody YTH53.1 (Thermo Fisher Scientific Cat# MA1-81489, RRID:AB_929140), and SDS were purchased from Thermo Fisher Scientific (Waltham, MA, USA).

Phase contrast microscopy was performed with an Olympus IX70 inverted fluorescence microscope (Olympus Life Science; Waltham, MA; RRID: SCR_018604). Immunofluorescence microscopy was performed with an EVOS FL Cell Imaging System fluorescence microscope (Thermo Fisher Scientific; Waltham, MA, USA). Viability assay fluorescence was read on a SpectraMax i3x microplate reader (Modular Devices; San Jose, CA, USA).

**Figure 3:**
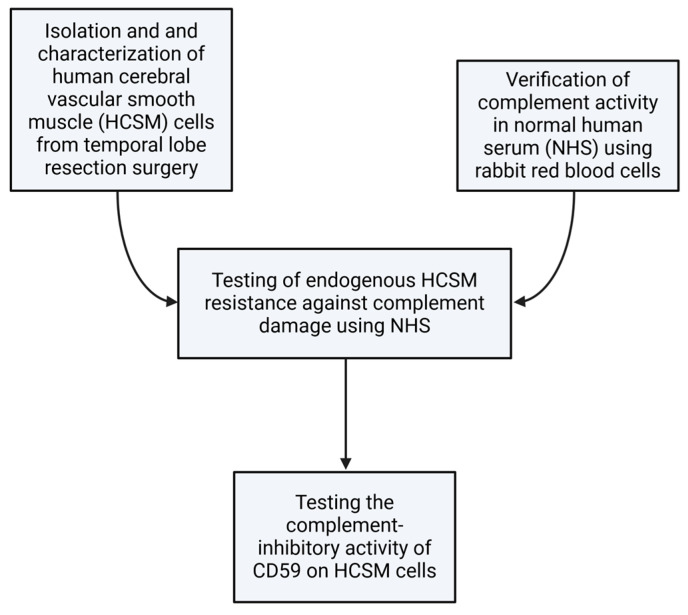
Visual summary of the methodological workflow. Created with BioRender.com.

The sequence of the methodological steps is represented in Figure 3 and described more fully in the succeeding subsections.

### 2.1. Cell Isolation and Culture

Brain tissues were obtained from a routine temporal lobe biopsy, performed at Loma Linda University Medical Center, as approved by the institutional IRB (#5170023). Per inclusion criteria, the individual donor was a non-prisoner within the age range of 18–75 years, previously diagnosed with medical intractable epilepsy and selected for neurosurgical intervention. Small blood vessels were manually isolated with forceps, washed, and sonicated to obtain primary human cerebral vascular (HCV) cells. The cells were incubated, expanded, and passaged in SM cell media supplemented with 2–5% *v*/*v* FBS, 100 units/mL penicillin, 100 µg/mL streptomycin, and SM cell growth supplement (50 µL/mL). Cells were cryopreserved in complete SM cell media supplemented with 40% *v*/*v* FBS and 10% *v*/*v* glycerol. Active cultures were kept incubated in 5% CO_2_ at 37 °C in complete SM cell media, which was changed every two or three days.

The HCV cell phenotype was identified to be HCSM by visual confirmation of the co-expression of alpha smooth muscle actin (αSMA) and desmin. Short tandem repeat analysis was carried out by the University of Arizona’s Genetics Core ((Facility RRID:SCR_012429) https://azgc.arizona.edu, accessed on 6 October 2021) and confirmed that the HCSM cells were genetically distinct from available cell lines.

### 2.2. Immunofluorescence Microscopy

Collagen-coated glass coverslips were placed in 6-well plates at 2–3 coverslips per well. HCV cells were seeded into the wells at a density that approximated 2 × 10^4^ cells per coverslip. Coverslips were fixed for 20 min with 4% paraformaldehyde in DPBS with calcium and magnesium (+Ca^++^/+Mg^++^) at 18 °C. Some coverslips were stored in DPBS +Ca^++^/+Mg^++^ at 4 °C for 1–2 weeks and the cells subsequently permeabilized with 0.2% triton X. All cells were blocked with 1% BSA in DPBS +Ca^++^/+Mg^++^. Then, the cover slips were rinsed three times. Primary and secondary antibodies were applied at concentrations suggested by the manufacturer, and incubated at the laboratory’s room temperature of 18 °C for 1 h each. Coverslips were mounted on glass microscope slides, allowed to cure overnight, and sealed with nail polish.

### 2.3. Alternative Complement Pathway Activity Test

Aliquots (2 mL) of normal human serum (NHS) were thawed in a <4 °C water bath and kept on ice to minimize complement protein degradation. Heat-inactivated serum (HIS) was created by heating thawed NHS in a 56 °C bead bath for 30 min [44,45]. Serum dilutions ranging from 1:8 to 1:16 were established by adding volumes of serum and gelatin veronal buffer to rabbit RBCs (1.5 × 10^7^ cells/mL). Gelatin veronal buffer or double-distilled water was added to rabbit RBCs to establish blank and total lysis controls, respectively. All RBC suspensions were subsequently incubated at 37 °C in 5% CO_2_ for 30 min with a gentle inversion halfway through. Cell suspensions were centrifuged at 1500× *g* for 5 min to sediment the RBCs, and the supernatants were transferred to a flat-bottomed 96-well plate at 100 µL/well as technical triplicates. To each well was added 100 µL of double-distilled water, and the absorbance values were measured at 540 nm. The percentage of total RBC lysis from each serum dilution was calculated with the following equation:

$$L=100\frac{\left(A_{t(av)}-A_{b(av)}\right)}{\left(A_{T(av)}-A_{b(av)}\right)},$$

where *L* = % lysis, *A_t_*_(*av*)_ = average test absorbance, *A_b_*_(*av*)_ = average blank absorbance, and *A_T_*_(*av*)_ = average absorbance after total lysis (adapted from Costabile, 2010) [46].

### 2.4. HCSM Cell Complement Resistance Assay

HCSM cells were seeded into flat-bottomed collagen type-1-coated 96-well plates at 16,700 cells/well and incubated at 37 °C in 5% CO_2_ to establish full confluence overnight. The following day, concentrations of normal or heat-inactivated human serum, ranging from 0 to 100% *v*/*v*, in SM cell media were applied to the wells. The wells were incubated at 37 °C in 5% CO_2_ for one hour, then washed three times with DPBS +Ca^++^/+Mg^++^. Cells were subsequently incubated with a 10% *v*/*v* solution of resazurin reagent in culture media for a period of two hours. The metabolic reduction of resazurin to resorufin was quenched by addition of SDS (3% *v*/*v* final), and the resultant solutions were stored in the dark at 4 °C, and analyzed within three days. Resorufin fluorescence intensity values were measured at 560/590 nm, and average fluorescence values were calculated for each serum concentration. The fluorescence values from cultures incubated with NHS were normalized to the fluorescence values from control cultures incubated with the same concentration of HIS to calculate the percentage difference in cellular viability. All assays were performed in triplicate and the resultant percent averages and standard errors were calculated. One-way ANOVA analysis and Tukey’s Honest Significant Difference post-hoc tests were applied to evaluate statistical significance.

### 2.5. CD59 Inhibition Assay

HCSM cells were seeded as described in the previous assay. Function-blocking anti-CD59 primary antibody YTH53.1 (αCD59) was applied to the wells at 0–50 µg/mL in 100 µL DPBS +Ca^++^/+Mg^++^ per well and incubated at 18 °C for 10 min before removal. Human serum was diluted to 80% *v*/*v* in SM cell media and added to the wells with biological triplicates per antibody concentration. The cells were incubated and washed, and the cellular viability was assessed with resazurin as described in the paragraph above. The average fluorescence values for each condition were normalized to that of the 0 µg/mL condition to calculate the percentage differences in cellular viability. These assays were performed in triplicate and the resultant percent averages and standard errors were calculated.

### 2.6. Statistical Analysis

One-way ANOVA analysis was performed using the Microsoft Excel (version 2405) software’s Data Analysis Tool. The subsequent Tukey’s Honest Significant Difference post-hoc test was performed using GraphPad Prism version 9.3.1.

## 3. Results

### 3.1. Generation of Primary HCSM Cells from Human Cerebral Vasculature

**Figure 4:**
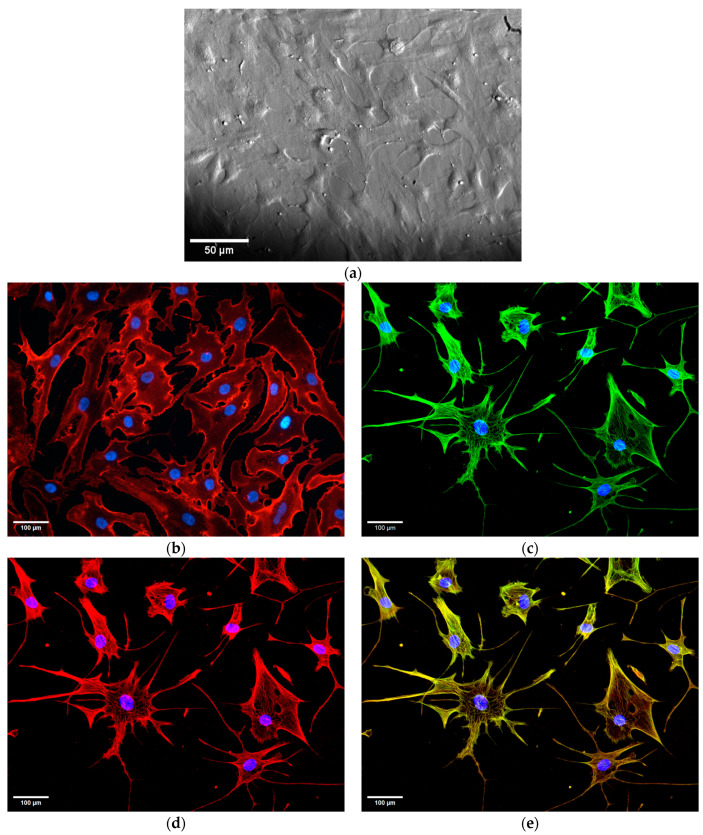
Visible morphology and marker protein expression in HCSM cells. (**a**) Phase contrast microscopy, 10× magnification. Fluorescence microscopy images of (**b**) CD59 surface expression (red), (**c**) αSMA expression (green), (**d**) desmin expression (red), (**e**) overlay of αSMA and desmin expression with extensive cytoskeletal overlap (yellow), with nuclear counterstain (blue) at 20× magnification. Microscopy images were adjusted using the Fiji distribution of ImageJ version 1.54f (NIH).

To explore how CD59 plays a role in primary HCSM cellular resistance to complement-dependent cytotoxicity, we first generated physiologically-relevant primary cells from a brain sample of a temporal lobe resection patient as described in the Materials and Methods section. As Figure 4a shows, the visible morphology of the HCV cells resembles the synthetic phenotype of SM cells as opposed to the contractile phenotype, as defined earlier [47]. We verified that all (100%) of the examined cells express external plasma membrane-bound CD59 (Figure 4b). The brighter fluorescence observed at the HCSM cell margins indicates higher CD59 expression in those areas. Cytoskeletal elements αSMA (Figure 4c) and desmin (Figure 4d) were both expressed and extensive, but incomplete overlap was observed between both proteins, confirming that double staining did not occur (Figure 4e) [48]. 

### 3.2. Functional Evaluation of CD59 in HCSM Cell Resistance to Complement Attack

**Figure 5:**
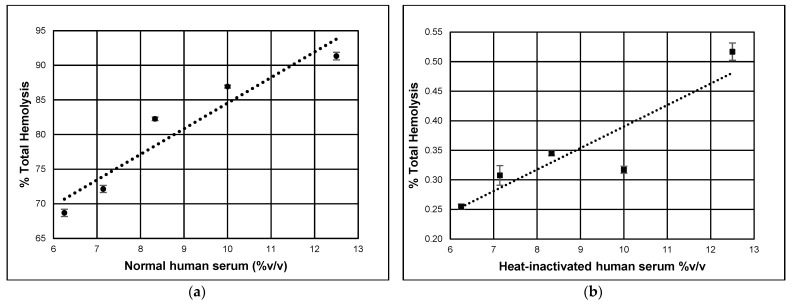
Alternative complement pathway activity in human serum. Dilutions of human serum were applied to rabbit red blood cells. Percentage of total hemolysis measures the cytolytic complement activity. (**a**) Normal human serum (y = 3.699x + 47.555; R^2^ = 0.9079). (**b**) Heat-inactivated human serum (y = 0.0364x + 0.0265; R^2^ = 0.816). Error bars represent +/− SE. Graphs were constructed using Excel, Microsoft 365 version 2405 (Microsoft).

CD59 contributes to complement attack resistance by preventing the formation of a complete MAC structure. To quantify the level of protection that CD59 imparts on primary HCSM cells, a source of complement proteins capable of generating functional MACs was required. To verify that the human serum used in these experiments can generate the necessary functional MACs, rabbit erythrocytes were utilized because they are vulnerable to cytolysis through spontaneous activation of the alternative complement pathway [49,50]. Heat-inactivated human serum acted as a negative control since complement proteins are sensitive to thermal denaturation [44,45]. Rabbit erythrocytes were subjected to a series of human serum concentrations, normal or heat-inactivated, and absorbance values were measured for liberated hemoglobin to determine the percentage of lysed cells. As Figure 5 shows, standard curves were established, demonstrating that the complement cascade is active in normal human serum (68.70–91.33% total hemolysis), whereas minimal complement activity is detected in the heat-inactivated serum (0.25–0.52% total hemolysis). The concentration of serum required to lyse 75% of the rabbit erythrocytes was calculated to be approximately 7.4% *v*/*v*.

**Figure 6:**
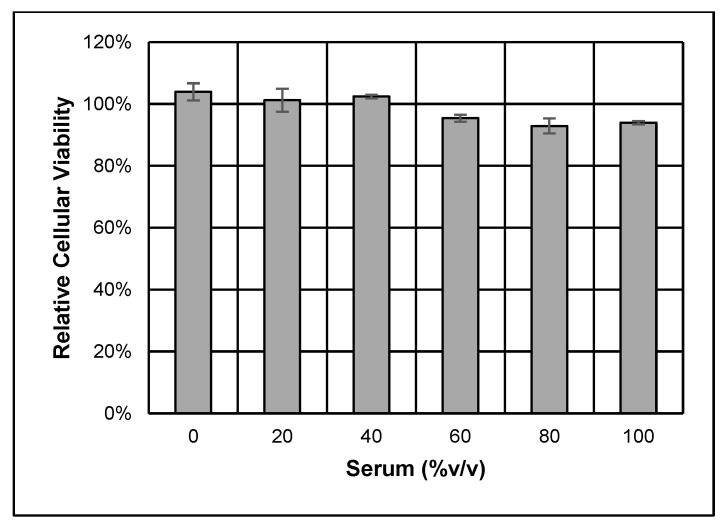
Endogenous HCSM cellular resistance to complement-dependent cytotoxicity. Cultured cell viabilities were measured by resazurin assay and then normalized to their respective HIS serum conditions to obtain relative percentages. Three separate assays were performed, with each condition examined in triplicate. One-way ANOVA testing and Tukey’s HSD post-hoc analysis showed no significant differences in outcome between conditions. Error bars are +/− SE. The graph was constructed using Excel, Microsoft 365 version 2405 (Microsoft).

Once the NHS was confirmed to be a source of functional complement, then the general HCSM cellular resistance was tested against complement-dependent cytotoxicity to determine how inherently vulnerable these cells are to the MAC. Cultured HCSM cells were challenged with up to the maximum concentration of normal human serum or heat-inactivated human serum. The relative viabilities of the challenged cell cultures were subsequently measured by their metabolic conversion of resazurin to fluorescent resorufin. As Figure 6 shows, HCSM cells proved resistant to complement-induced cytotoxicity. The approximately 8.4% decrease in HCSM cell viability, observed under the 60–100 % *v*/*v* NHS conditions, did not reach statistical significance according to Tukey’s post-hoc test.

**Figure 7:**
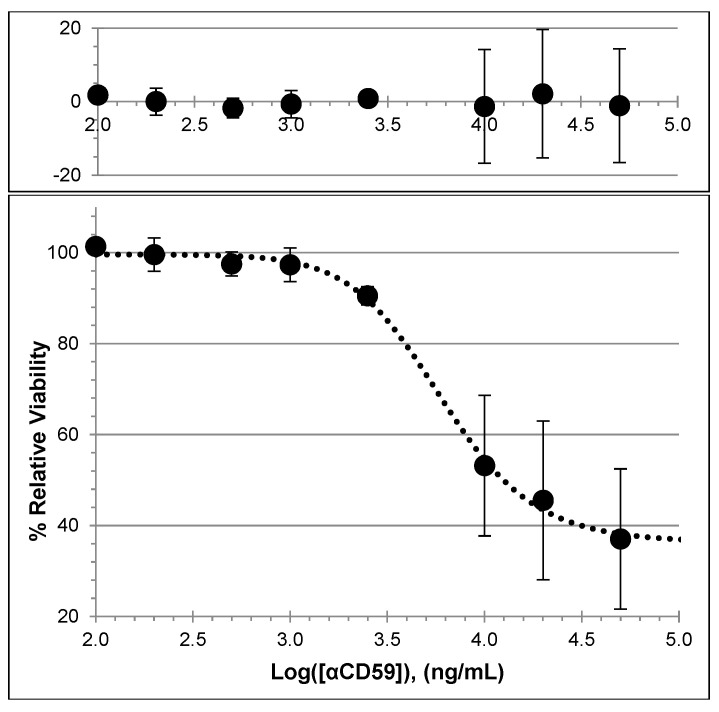
HCSM cellular resistance to complement decreases following treatment with αCD59. Main panel: The αCD59 dose–response fitted to a four-factor sigmoid function. Upper panel: The residuals of measured cell viability compared to calculated values using the sigmoid function. All assays were performed in triplicate. Error bars are +/− SE. Graphs were constructed using Excel, Microsoft 365 version 2405 (Microsoft). Based on the fitted parameters, the cytotoxic EC_50_ of αCD59 is 6.0 µg/mL. The fitted viability at saturating αCD59 levels is 36.2%.

The degree to which endogenous CD59 surface expression protects HCSM cells from complement-dependent cytotoxicity was evaluated by blocking the CD59 with increasing concentrations of αCD59 [51,52]. Then, the blocked HCSM cells were challenged with normal human serum (80% *v*/*v*) and the relative viabilities were subsequently measured by their metabolic conversion of resazurin to fluorescent resorufin to establish the dose–response curve seen in Figure 7. The non-linear regression fit of the data produced the following equation, in which *X* is the Log(αCD59 concentration) in ng/mL and *Y* is the percentage of cellular viability relative to that of untreated HCSM control cells:

$$Y=36.2+\frac{99.6-36.2}{{1+(\frac{X}{3.78})}^{15.86}}$$

## 4. Discussion

We identified our isolated primary human cerebral vascular cells as HCSM cells by their visible morphology and endogenous expression of both αSMA and desmin (Figure 4c,d). The visible morphology appeared to be consistent with the SM cell synthetic phenotype. In contrast with the spindle-shaped contractile phenotype characteristic of SM cells in healthy blood vessels, the synthetic phenotype is a less differentiated form that is associated with SM cell migration, proliferation, and post-insult vessel repair [47]. While αSMA is also present in myofibroblasts, a cell type that could hypothetically be extracted and cultured by accident, the muscle cell marker desmin is only weakly expressed in one subtype of myofibroblast [53,54]. The strong expression of both cell markers is consistent with these cells being physiologically SM. Primary HCSM cells were utilized instead of immortalized cell lines, which have undergone genetic changes that may alter their behavior. The primary smooth muscle cells were sourced from the cerebral vasculature rather than the aorta in order to maximize the physiological relevancy.

Nucleated cells are not lysed by the MAC unless multiple complete channels are formed across the plasma membrane. Complement-induced damage is resisted through increased cell proliferation, the inhibition of apoptosis, and the elimination of terminal complexes from the plasma membrane [55,56]. By contrast, non-nucleated erythrocytes are relatively vulnerable to the action of the terminal complement complex, as only a single completed complement channel is required to initiate cytolysis [56,57]. The measurement of complement-driven hemolysis is a standard method to determine the relative levels of complement pathway activity present in human serum. Rabbit erythrocytes are vulnerable to spontaneous activation of the alternative complement pathway, and therefore are used to determine the percentage of human serum required to lyse 75% of the suspended rabbit erythrocytes. Comparison of the respective percentages of hemolysis of NHS and HIS (Figure 4) confirms that the normal human serum used in these studies contains the active set of complement proteins required to form functional membrane attack complexes.

Cultured HCSM cells exhibiting the synthetic phenotype appear mostly resistant against high levels of complement until a sufficient proportion of their surface CD59 molecules are bound and inactivated by an anti-CD59 antibody (Figure 5 and Figure 6). It is worth noting the possibility that a complement attack on the HCSM cells may have proceeded through different pathways depending on the assay. HCSM cells that were not blocked with αCD59 could only have been subjected to the alternative pathway because antibodies are required to activate the classical pathway, whereas HCSM cells blocked with αCD59 may have been affected through both the alternative and classical pathways [14]. The IgG_2_ antibody subclass to which αCD59 belongs is overall less efficient at activating the classical pathway than either the IgG_1_ or IgG_3_ subclasses, but IgG_2_ effectiveness increases with increasing antigen concentration [58,59,60,61]. It is likewise possible that cells blocked with αCD59 may have experienced some alterations in cellular function. Consequently, it is possible that cross-linking CD59 with antibodies may lead to apoptosis [62], though we did not specifically investigate to what degree such an effect might contribute to the progressive loss of cellular viability.

Thus, under normal physiological conditions, the endogenous CD59 expression levels of HCSM cells are sufficient to protect these cells from complement-driven cytolysis. Because our experiments utilized human serum as the complement protein source, levels of complement did not exceed the range found in whole blood. Therefore, we currently cannot predict whether excessive complement levels might overwhelm the endogenous CD59, or whether the HCSM cells can upregulate CD59 to compensate.

The apparent saturation of CD59 with αCD59 did not produce fluorescence values that indicate zero cellular viability, suggesting that the complete inhibition of CD59 is not sufficient to eliminate all HCSM cells within a culture through complement-mediated cytotoxicity. This may be due to the protective action of other plasma membrane-bound complement regulators, such as CD55 and CD46, in conjunction with the self-repair mechanisms employed by nucleated cells.

Our study is constrained by its reliance on a sole source for the primary HCSM cells. The gender, ethnic background, and exact age of the donor are unknown. These factors may affect the exact levels of CD59 expression observed. To confirm the validity and generalizability of our findings, future investigations should include such variables.

Taken together, these data indicate that CD59 expression and functionality are critical aspects of endogenous HCSM cellular defense against complement-mediated cytotoxicity. Further, this suggests that the artificial enhancement of CD59 expression may be an effective means of protecting HCSM cells if the normal expression declines or if complement system activity becomes aberrantly high. This approach would leave the upstream complement cascade intact, avoiding potential issues such as increased susceptibility to bacterial infection and interference with certain neurologic processes [28]. The insertion of additional CD59 gene copies into the nuclear DNA, for example, has been shown to increase resistance to complement-induced cytolysis in a number of cell types [38,63,64].

An alternative or supplementary approach might be to enhance CD46 and CD55 expression, though their respective efficacies when compared to CD59 are currently unknown for this cell type. The degree to which alternative complement regulatory proteins and cellular repair mechanisms mitigate complement damage to HCSMs remains to be further elucidated. In addition, both proteins interrupt the complement cascade at earlier points, and may risk exacerbating the issues discussed above. Another potential alternative is to employ the therapeutic anti-complement antibodies eculizumab and ravulizumab, which are primarily used to treat paroxysomal nocturnal hemoglobunuria, a disorder in which the red blood cells exhibit a deficiency of CD59 and so are vulnerable to MAC. The antibodies are generally well tolerated, but increase the risk of meningococcal infections, so patients must be vaccinated prior to treatment [65,66,67].

## Data Availability

Data are available upon request.
